# Multilayer and Multiplex Networks: An Introduction to Their Use in Veterinary Epidemiology

**DOI:** 10.3389/fvets.2020.00596

**Published:** 2020-09-04

**Authors:** Amy C. Kinsley, Gianluigi Rossi, Matthew J. Silk, Kimberly VanderWaal

**Affiliations:** ^1^Department of Veterinary Population Medicine, College of Veterinary Medicine, University of Minnesota, St. Paul, MN, United States; ^2^Roslin Institute and Royal (Dick) School of Veterinary Sciences, University of Edinburgh, Edinburgh, United Kingdom; ^3^Centre for Ecology and Conservation, University of Exeter Penryn Campus, Penryn, United Kingdom; ^4^Environment and Sustainability Institute, University of Exeter, Penryn, United Kingdom

**Keywords:** network analysis, multilayer networks, animal movement, pigs, transmission, infectious disease

## Abstract

Contact network analysis has become a vital tool for conceptualizing the spread of pathogens in animal populations and is particularly useful for understanding the implications of heterogeneity in contact patterns for transmission. However, the transmission of most pathogens cannot be simplified to a single mode of transmission and, thus, a single definition of contact. In addition, host-pathogen interactions occur in a community context, with many pathogens infecting multiple host species and most hosts being infected by multiple pathogens. Multilayer networks provide a formal framework for researching host-pathogen systems in which multiple types of transmission-relevant interactions, defined as network layers, can be analyzed jointly. Here, we provide an overview of multilayer network analysis and review applications of this novel method to epidemiological research questions. We then demonstrate the use of this technique to analyze heterogeneity in direct and indirect contact patterns amongst swine farms in the United States. When contact among nodes can be defined in multiple ways, a multilayer approach can advance our ability to use networks in epidemiological research by providing an improved approach for defining epidemiologically relevant groups of interacting nodes and changing the way we identify epidemiologically important individuals such as superspreaders.

## Introduction

The use of social network analysis and modeling in epidemiology has significantly enhanced our understanding of pathogen transmission dynamics in populations with heterogeneous contact (1–3). Network analysis gained traction with the field of veterinary epidemiology over a decade ago and has often been applied to livestock and wildlife populations in an attempt to unravel the impact of contact heterogeneity on the spread of pathogens (4–9). These advancements have led to greater knowledge surrounding potential risks for disease spread, which ultimately support decision-making pertaining to resource allocation for surveillance, management, and control strategies (10–12).

**Figure 1:**
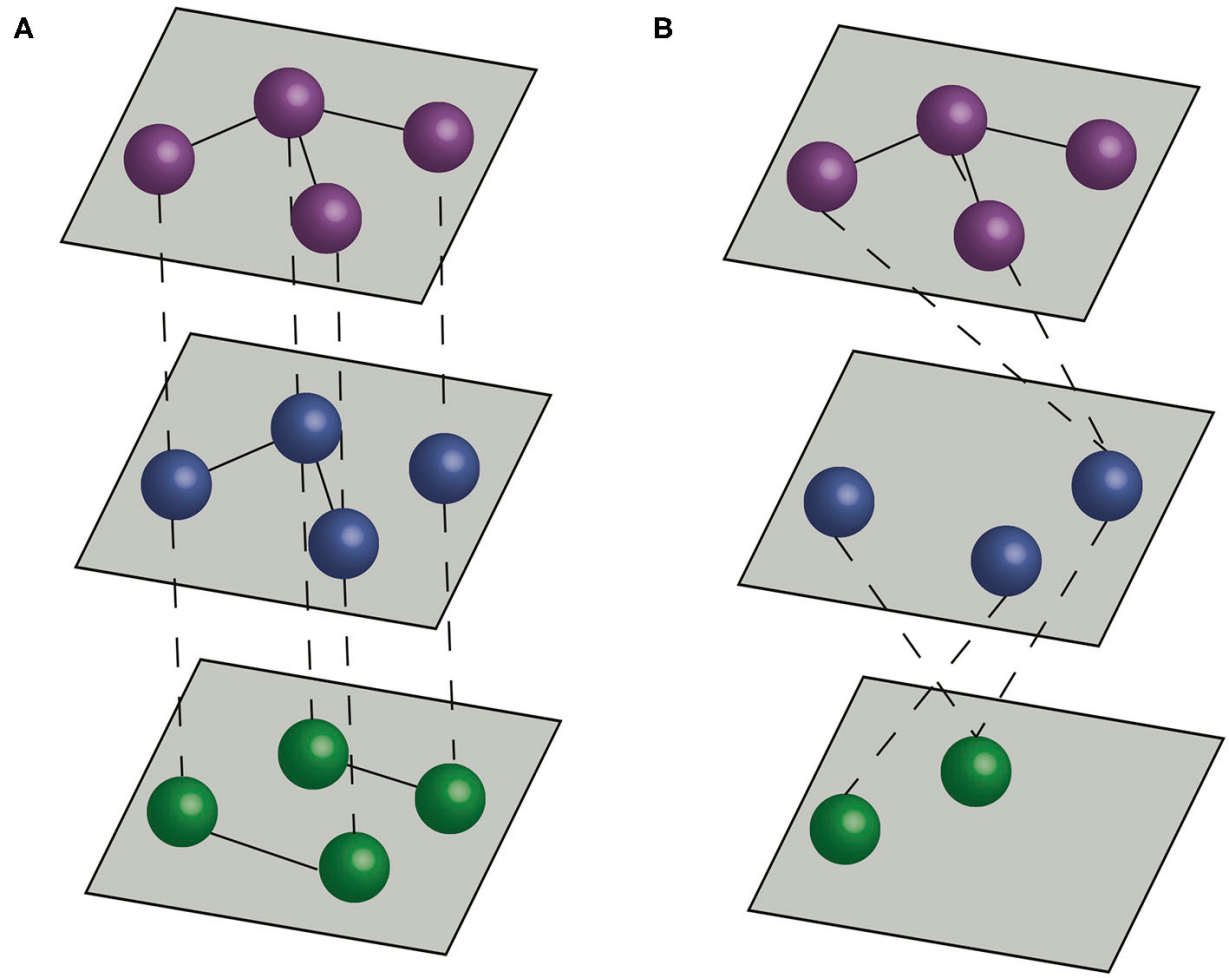
Multilayer networks. Dashed lines represent interlayer connections, and solid lines represent intralayer connections. **(A)** A multiplex network formed by three layers, with interlayer edges connecting the same individual across layers. **(B)** An interconnected network formed by three layers, with interlayer edges connecting different individuals across layers.

Although social network approaches provide a robust framework to study a variety of systems, they can fall short of capturing complexity associated with interactions that are commonly considered in veterinary epidemiology. In many contexts considering the role of different types of contact (e.g., different types of social interactions, different types of movement between farms or interactions between different species) can have a significant impact on our understanding of how infectious diseases spread (13–15). Multilayer networks facilitate such an approach by including multiple network layers to more explicitly represent features of natural systems (16, 17). In traditional contact networks, or disease-relevant social networks, nodes represent individuals or populations, and edges represent disease-relevant contacts between the nodes. In multilayer networks, nodes are organized into layers, and edges can connect nodes in the same layer (*intralayer edges*) or nodes in different layers (*interlayer edges*) (Figure 1).

The separation of layers within the multilayer framework allows for the coupling of dynamical processes across and within layers and has consequently revealed phenomenon unattainable through traditional network representations (18). For example, the multilayer network framework has been used to capture epidemiological processes contributing to our understanding of the influence of information spread (19, 20), social support on infectious disease transmission (19–22), the role of different species in multi-host infections (23, 24), and the role of different modes of transmission in infectious disease dynamics (25, 26).

The purpose of this review is to highlight the potential uses of multilayer networks in veterinary epidemiology. The review is divided into four main sections. The first describes key terms and techniques commonly used in multilayer network analysis. We then review the use of multilayer models in human and veterinary epidemiology. We provide an example using U.S. swine networks representing contact through swine shipments and spatial proximity. Finally, we discuss important considerations when using the approach in an epidemiological context and outline some key research questions that multilayer network approaches will help veterinary epidemiologists address.

## Multilayer Network Methodology

### Terminology

The power of multilayer networks lies in their flexibility to characterize multiple types of interactions not possible using a traditional monolayer network approach. In monolayer networks, *edges* (or links) represent connections between *nodes* that can be directed or undirected. For example, networks may describe social associations (undirected edges) among wild animals (each individual being a node) or movements (directed edge) from one farm to another (each farm being a node). Multilayer networks also consist of *nodes and edges*, but the nodes exist in separate *layers*, representing different forms of interactions, which connect to form an *aspect* (16, 17). Aspects, or stacks of layers, can be used to represent different types of contacts, spatial locations, subsystems, or points in time. The edges between nodes in the same layer of an aspect are called *intralayer connections*, whereas edges between nodes in different layers are *interlayer connections* (17, 18, 23).

There are two main types of multilayer networks, *multiplex networks* and *interconnected networks* (17, 27). In *multiplex networks*, interlayer edges can only connect nodes that represent the same actor in different layers. Therefore, multiplex networks typically represent sets of interactions between the same (or a similar set) of entities (e.g., individuals, farms). In *interconnected networks*, interlayer edges can connect between different actors, and therefore different layers typically represent different entities (e.g., individuals of different species, or farms in different production systems) (Figure 1). Thus, the structure of interlayer edges can be used to distinguish different types of multilayer network. When interlayer edges can only link nodes to nodes representing the same entity (the same individual animal or farm) in different layers, the network is classified as a *multiplex network* (28, 29). When interlayer edges can link nodes representing one entity to nodes connecting others in different layers then the network is classified as an *interconnected network*.

*Multi-relational network*s are an example of a multiplex network (30). In multi-relational networks, layers may represent the same population of individuals but with different forms of contact, which is advantageous for representing different modes of transmission. For example, one layer may represent direct contact in which edges represent the shipment of animals between farms, and the other may represent indirect contact through edges representing a shared source of feed. Another example of a multiplex network is a *temporal network* in which each node is connected to itself over discrete layers that represent time periods, but the connections between individuals within a layer represent interactions captured during that duration of time (16, 17, 30). Understanding variation in the temporality of disease processes can be critical to the application of intervention activities as well as providing useful information surrounding potential sources of infection.

### Extending Centrality Measures to Multilayer Networks

*Centrality* is often used as a measure of an individual's importance in a network and as proxy for its role in the transmission of infection (31). Measures of centrality include local measures such as *degree* and *strength* that take into account only immediate neighbors in the network (31), global measures such as *closeness* and *betweenness centrality* that take into account the entire network structure (31), and intermediate measures such as *eigenvector* (32)*, Katz and PageRank centralities* (33) that account for some indirect connections when calculating the influence of an individual. In monolayer networks, centrality measures have been used to identify individuals with disproportionately large numbers of contacts that serve as potential super-spreaders (34) or can be crucial cut-points (35) or capacitors (36) in the spread of infection.

The multilayer network approach allows for flexibility to capture an individual's engagement in contact across a variety of disease-relevant contexts by extending the suite of centrality measures to consider interactions within and across layers. *Multidegree* is a vector of the connectedness of an individual in each layer of a multiplex network, and the same vector of centralities can be used for other measures (37). Quantifying the centrality of nodes for multiple layers makes it possible to consider how its connectedness is distributed across layers and can provide nuance in identifying which individuals might be most important to the spread of infection through different transmission modes. *Versatility* provides a single measure of a node's importance across multiple layers and considers the full multilayer structure (38). Various versatility metrics can be implemented for *betweenness, eigenvector*, and *PageRank* centralities (39), and can be calculated using the *MuxViz* software (40). For centrality metrics that are based on paths within a network, such as betweenness, it readily apparent how a multi-layer index that allows a path to traverse the network via several different layers could better capture a node's importance when there are multiple transmission modes. Individuals or farms that are not especially well connected in any one layer may have the highest versatility if they are well connected across multiple layers.

### Mulitplex Neighborhoods and Relevance

It is also possible to calculate the importance of particular layers within multiplex networks. The *neighborhood* of an individual in a single or specified set of layers is the number of actors connected to an actor (or node) in that layer (or set of layers). From this it possible to calculate the *exclusive neighborhood*, the number of nodes directly connected to a focal node only in that layer or set of layers, and the *connective redundancy* of a layer (or set of layers) which is $1-\frac{neighborhood}{total\;degree}$. Finally, the *relevance* of a layer is the percentage of neighbors present in a specified set of layers, and the *exclusive relevance* is the percentage of neighbors only present in that set of layers. These measures can be calculated using the *multinet* package (41) in R (42). They can be used to provide some indication of the role of different layers in a multiplex network, and in epidemiological context would be most useful in identifying layers that are especially important to transmission, especially in spreading infection to parts of the population that are less well connected in general.

### Extending Community Detection Methods to Multilayer Networks

Often, nodes within a network are clustered. Nodes that are directly connected are more likely to share mutual connections (*transitivity*) and networks can often be subdivided into *communities* (or modules) in which within-community connections are much more frequent than connections between individuals in different communities. The strength of these subdivisions is measured using *modularity* and can have important implications for disease transmission. For example, networks with higher levels of *modularity* tend to have a slower spread of infectious disease (43). Communities in multilayer networks are defined in a similar manner, but can account for variation in connectivity across layers (44). Frequently used examples of multilayer community-detection algorithms include multislice modularity maximization (44, 45), which maximizes the modularity quality over the network partitions by comparing the total edge weights in an observed network to the total expected edge weights in a “null network” (45), and Infomap, which maximizes the map equation by identifying cluster structures in a network and minimizing the description length of a random walker on a network (39, 46). Community detection in multilayer networks might be useful in taking into account multiple transmission routes (i.e., different types of contact) while identifying epidemiologically relevant clusters of individuals that could represent single units for management interventions. It could also be used to identify clusters of individuals that play a key role in disease spread through multiple routes of transmission, but at different time points.

### Compartmental Models on Multilayer Networks

Mathematical modeling has long been an important tool in veterinary epidemiology, principally in the form of compartmental models (47). These approaches model the transition of individuals between disease states with examples including the widely used SI (susceptible-infected), SIR (susceptible-infective-recovered), SIS (susceptible-infective-susceptible), and SEIR (susceptible-exposed-infectious-recovered) models. In general, compartmental models on networks tend to be individual-based (5, 48, 49), but veterinary epidemiological studies have developed population-based network models (50), which are often more suitable for studying livestock populations. Compartmental models have already been applied to study the spread of infectious disease in multilayer networks (21, 22, 51, 52), and can frequently provide additional insights into infectious disease dynamics. Methods for modeling infectious disease transmission on multilayer networks are similar to those developed for compartmental metapopulation models of disease spread but generally support higher levels of complexity than metapopulation models, as they allow for the integration of multiple modes of contact within and between population and other interconnecting processes (53). It has been shown that, when interlayer edges connect individuals in different, discrete populations and intralayer edges connect individuals within each population, certain distributions of interlayer vs. intralayer edges can cause outbreaks in the system as a whole which would not occur in any single population (layer) within the system. Further, under certain conditions, the epidemic threshold of the whole system may be smaller than the epidemic threshold of its parts (54). These additional insights can be important in exploring the effects of interventions strategies aimed at different subpopulations or the effects of multiple spreading processes, such as disease awareness or vaccination behavior (22, 55), which can continue to advance our understanding of the influence of complex contact structures on infectious disease dynamics.

## Previous Uses of Multilayer Networks in Epidemiology

The scientific study of multilayer networks is a burgeoning area of research, particularly the development of theoretical epidemiological models and its application to human epidemiology. Although its use in veterinary contexts is still limited, here we outline key areas of research that have been pursued in theoretical and empirical studies (in both humans and animals) and highlight how multilayer networks might be applied to veterinary epidemiology.

### Different Routes of Infection

Multilayer networks can be usefully applied in contexts where a pathogen can be transmitted through multiple modes or pathways of infection (12), as the multiplex approach provides a framework to account for multiple transmission probabilities. Considering the presence of multiple transmission modes can influence the efficacy of targeted interventions, particularly if nodes were traditionally targeted according to their degree in only one layer (25, 26, 56). This has implications for situations where data, networks, and resultant optimal control strategies are only available for one mode of transmission, leading to overconfidence in the efficacy of control.

In the context of veterinary epidemiology, animal movements are typically considered the most effective transmission mode between farms (direct contacts) (57). However, other infection mechanisms might play an important role such as wind-borne spread and fomites disseminated through contaminated clothes, equipment, and vehicles by personnel (indirect contacts) (58–60). Ignoring one mode of transmission could lead to inaccurate farm risk predictions and ineffective targeted surveillance. This has been demonstrated in a network analysis that considered both direct (cattle movements) and indirect (veterinarian movements) contacts to reveal that indirect contact, despite being less efficient in transmission, can play a major role in spread of a pathogen within a network (13).

In another example, Stella et al. (51) used an “ecomultiplex model” to study the spread of *Trypanosoma cruzi* (cause of Chagas disease in humans) across different mammal species. This pathogen can be transmitted either through invertebrate vectors (Triatominae or kissing bugs) or through predation when a susceptible predator feeds on infected prey or vectors. Thus, their model included two ecological/transmission layers: the food-web and vector layers. Their results showed that studying the multiplex network structure offered insights on which host species facilitate parasite spread, and thus which would be more effective to immunize in order to control the spread. At the same time, they showed how, in this system, when parasites spread occurs primarily through the trophic layer, immunizing predators hampers parasite transmission more than immunizing prey.

Furthermore, multilayer network analysis can help differentiate between different types of social interactions that may lead to disease transmission. For example, sex-related dynamics of contact networks can have important implications for disease spread in animal populations, as seen in the spread of *Mycobacterium bovis* in European badgers (*Meles meles*) (61). The authors constructed an interconnected network that distinguished male-male, female-female, and between-sex contacts recorded during proximity loggers. Inter-layer between-sex edges and edges in the male-male layer were more important in connecting groups into wider social communities, and contacts between different social communities were also more likely in these layers.

### Dynamics of Coupled Processes—the Spread of Two Pathogens

Another application of multilayer networks in epidemiology is to model the concurrent propagation of two entities through a network, such as two different pathogens co-occurring in the same population or the spread of disease awareness alongside the spread of infection. In both scenarios, the spread of one entity within the network interacts with the spread of the other, creating a coupled dynamical system. A multiplex approach can allow for each coupled process to spread through a network that is based on the appropriate type of contact for propagation (i.e., contact networks involved in pathogen transmission vs. interaction or association networks that allow information to spread). In the case of two infectious diseases concurrently spreading through a network, a multiplex approach can be particularly useful if infection of a node by pathogen *A* alters the susceptibility to pathogen *B*, or if coinfection of a node influences its ability to transmit either pathogen. For example, when infection by one pathogen increases the likelihood of becoming infected by another pathogen, it could theoretically facilitate the spread of a second pathogen and thus alter epidemic dynamics (62). This type of dynamic is likely to widespread in wild and domestic animals due to the importance of co-infection in affecting infectious disease dynamics by influencing the replication of pathogens within hosts (63). However, when there is competition or cross-immunity, the spread of one pathogen could reduce the spread of a second pathogen (64). For example, this type of dynamic could be expected for pathogens strains characterized by partial cross-immunity, such as avian influenza (65), or microparasite-macroparasite coinfections in which infection with one parasite reduces transmission of a second, such as infection with gastrointestinal helminths reducing the transmission of bovine tuberculosis in African buffalo (*Syncerus caffer*) (66). Similar “within-node” dynamics could be important at a farm-level in livestock movement networks. For example, the detection of a given pathogen infection in a farm might cause it to be quarantined, thus reduce its susceptibility and ability to transmit other pathogen infections.

### Dynamics of Coupled Processes—Interactions Between Transmission Networks and Information/Social Networks

For coupled processes involving a disease alongside a social process (i.e., spread of information or disease awareness), we might expect that the spread of the pathogen will be associated with the spread of disease awareness or preventative behaviors such as mask-wearing, and in these cases theoretical models suggest that considering the spread of disease awareness can result in reduced disease spread (67). A model was presented by Granell et al. (19), which represented two competing processes on the same network: infection spread (modeled using a Susceptible-Infected-Susceptible compartmental model) coupled with information spread through a social network (an Unaware-Aware-Unaware compartmental model). The authors used their model to show that the timing of self-awareness of infection had little effect on the epidemic dynamics. However, the degree of immunization (a parameter which regulates the probability of becoming infected when aware) and mass media information spread on the social layer did critically impact disease spread (19). A similar framework has been used to study the effect of the diffusion of vaccine opinion (pro or anti) across a social network with concurrent infectious disease spread. The study showed a clear regime shift from a vaccinated population and controlled outbreak to vaccine refusal and epidemic spread depending on the strength of opinion on the perceived risks of the vaccine. The shift in outcomes from a controlled to uncontrolled outbreak was accompanied by an increase in the spatial correlation of cases (20). While models in the veterinary literature have accounted for altered behavior of nodes (imposition of control measures) as a result of detection or awareness of disease (68), it is not common for awareness to be considered as a dynamic process that is influenced by how each node has interacted with the pathogen (i.e., contact with an infected neighbor). For example, the rate of adoption of biosecurity practices at a farm, such as enhanced surveillance, use of vaccination, or installation of air filtration systems, may be dependent on the presence of disease in neighboring farms or the farmers' awareness of a pathogen through a professional network of colleagues.

There is also some evidence that nodes that are more connected in their “social support” networks (e.g., connections with family and close friends in humans) can alter network processes that result in negative outcomes, such as pathogen exposure or engagement in high-risk behaviors (22). In a case based on users of injectable drugs, social connections with non-injectors can reduce drug-users connectivity in a network based on risky behavior with other drug injectors (69). In a model presented by Chen et al. (22), a social-support layer of a multiplex network drove the allocation of resources for infection recovery, meaning that infected individuals recovered faster if they possessed more neighbors in the social support layer. In animal (both wild and domesticated) populations, this concept could be adapted to represent an individual's likelihood of recovery from, or tolerance to, infection being influenced by the buffering effect of affiliative social relationships (70). For domestic animals, investment in certain resources at a farm level could influence a premise's ability to recover (e.g., treatment) or onwards transmission of a pathogen (e.g., treatment or biosecurity practices). Sharing of these resources between farms could be modeled through a “social-support” layer in a multiplex, for example, where a farm's transmissibility is impacted by access to shared truck-washing facilities.

### Multi-Host Infections

Multilayer networks can be used to study the features of mixed species contact networks or model the spread of a pathogens in a host community, providing important insights into multi-host pathogens (12). Scenarios like this are commonplace at the livestock-wildlife interface and therefore the insights provided could be of real interest to veterinary epidemiology. In the case of multi-host pathogens, intralayer and interlayer edges represent the contacts between individuals of the same species and between individuals of different species, respectively. They can therefore be used to identify bottlenecks of transmission and provide a clearer idea of how spillover occurs. For example, Silk et al. (24) used an interconnected network with three layers to study potential routes of transmission in a multi-host system. One layer consisted of a wild European badger (*Meles meles)* contact network, the second a domesticated cattle contact network, and the third a layer containing badger latrine sites (potentially important sites of indirect environmental transmission). No intralayer edges were possible in the latrine layer. The authors demonstrated the importance of these environmental sites in shortening paths through the multilayer network (for both between- and within-species transmission routes) and showed that some latrine sites were more important than others in connecting together the different layers. Pilosof et al. (23) presented a theoretical model, labeling the species as focal (i.e., of interest) and non-focal, showing that the outbreak probability and outbreak size depend on which species originates the outbreak and on asymmetries in between-species transmission probabilities.

Similar applications of multilayer networks (see Supplementary Material) could easily be extended to systems where two or more species are domesticated animals, as well. Examples of these could be the study of a pathogen such as Bluetongue virus, which affects both cattle and sheep (71), or foot-and-mouth disease virus, which infects cattle, sheep, and pigs (60). In such cases, each species can be represented by a different level in the network, and interlayer edges are made possible as a result of mixed farms (i.e., cattle and sheep), different species from different farms grazing on the same pasture, or for other types of indirect contacts such as the sharing equipment or personnel.

Overall, multilayer approaches provide an elegant way to analyze cross-species transmission and spillover, including for zoonotic pathogens across the human-livestock-wildlife interface. They can be used to simultaneously model within-species transmission, identify heterogeneities among nodes in their tendency to engage in between-species contacts relevant for spillover and spillback, and better predict the dynamics of spread prior and subsequent to cross-species transmission events, which may contribute to forecasting outbreaks in target species. Measures of multilayer network centrality in this instance could be used to extend the superspreader concept into a community context; individuals that are influential in within-species contact networks and possess between-species connections might be predicted to have a more substantial influence on infectious disease dynamics in the wider community.

## Case Study: Multiplex Networks in the U.S. Commercial Swine Industry

**Figure 2:**
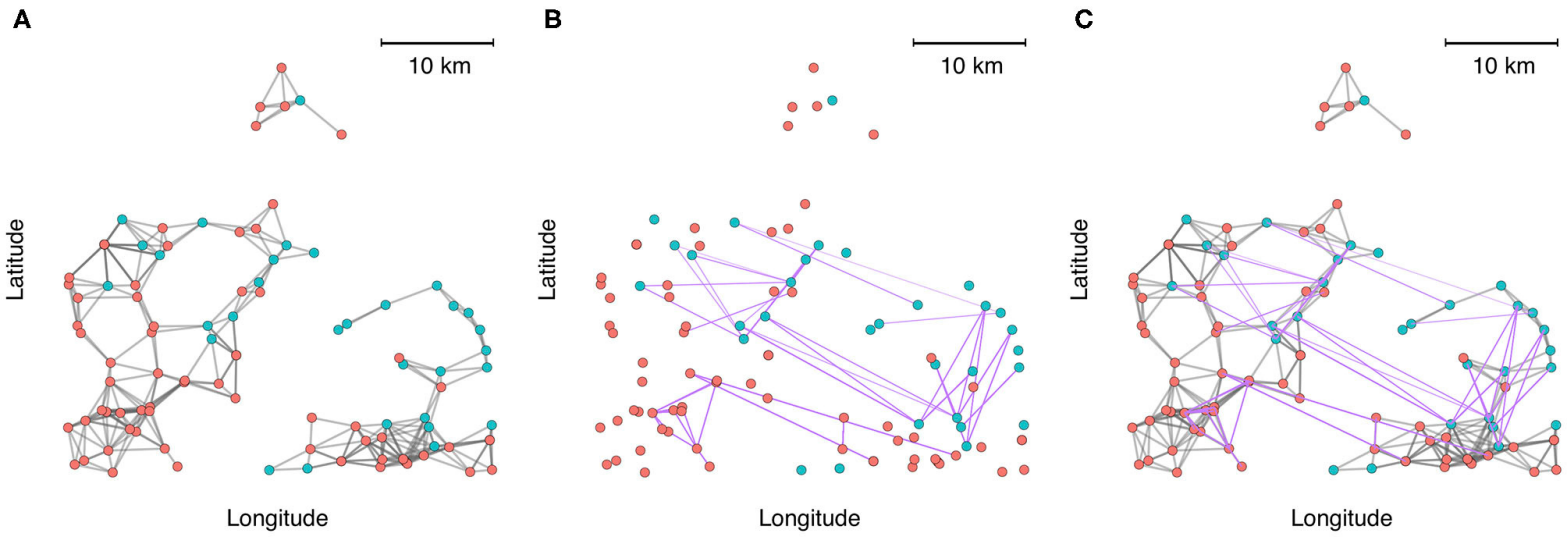
A zoomed-in subset of 100 farms plotted into geographic space, where node color represents each production system. **(A)** Connections between farms based on spatial proximity (<5 km). **(B)** Connections between farms based on animal movements. **(C)** Aggregate visualization of the spatial proximity and movement network, where edges are colored according to the type of contact (gray = spatial proximity, purple = animal movement).

To demonstrate the utility and application of multilayer network analysis, we provide an example from the commercial swine industry in the United States. Our objective is to cement the concepts presented in this review with a real-world example, and to demonstrate how a multi-layer approach can enhance insights on the identity of high-risk nodes (highly connected farms that have greater exposure or are potential super-spreaders) and the architecture and modularity of networks when multiple modes of contact are considered. In this example, we calculate centrality metrics and identify communities using data from 1,544 farms belonging to two swine companies from production systems that have been previously described (72). Both companies are vertically integrated, meaning that different phases of production occur at different farms (gestation and farrowing at *sow farms*, rearing of weaned piglets at *nursery farms*, and fattening pigs for the market at *finishing farms*). We created a multiplex network with two layers to account for multiple modes of transmission-relevant contact. Intralayer edges in one layer consisted of animal movement between farms as it is a known pathway of pathogen transmission between farms. The second layer consisted of predicted contacts arising from spatial proximity (threshold at < 5 km), because it has also been postulated that local area spread occurs via windborne spread or indirect contacts, such as shared personnel, trucks or equipment, for several important swine diseases (58, 73). For example, spatial proximity networks based on a 5 km threshold have been shown to be associated with the occurrence of porcine reproductive and respiratory syndrome virus (72). There are additional nuances to both spatial proximity (e.g., wind direction, climatic factors, vegetation (74), and animal movements (temporality, directed vs. undirected, etc.) that should be accounted for in a rigorous analysis of transmission within swine systems, but we have simplified these to create a clearer conceptual illustration of multi-layer networks. From an initial visual assessment of Figure 2, it is apparent that either layer alone would misrepresent connectivity patterns. Spatial proximity overestimates the fragmentation of the network across space, while animal movements underrepresent local connections.

We quantified the centrality of each node in the multiplex network, in each single-layer network, and the overall aggregated network using MuxViz v2.0.1 (40). In this analysis, we focused on degree, strength, and eigenvector centrality. Because the spatial proximity network was denser than the movement network, we re-scaled the edge weights such that the sum weight of all edges was equal to one in both networks. This helped ensure that the spatial proximity layer, which had higher density, was not excessively dominant over the movement layer which contained many fewer edges. In practice, the relative weighting of edges in different layers should be subject to a sensitivity analysis or tested with data (see *Points of considerations* section below), as this choice can influence multilayer metrics and communities. However, we used a simple re-scaling approach here to demonstrate multilayer concepts.

**Figure 3:**
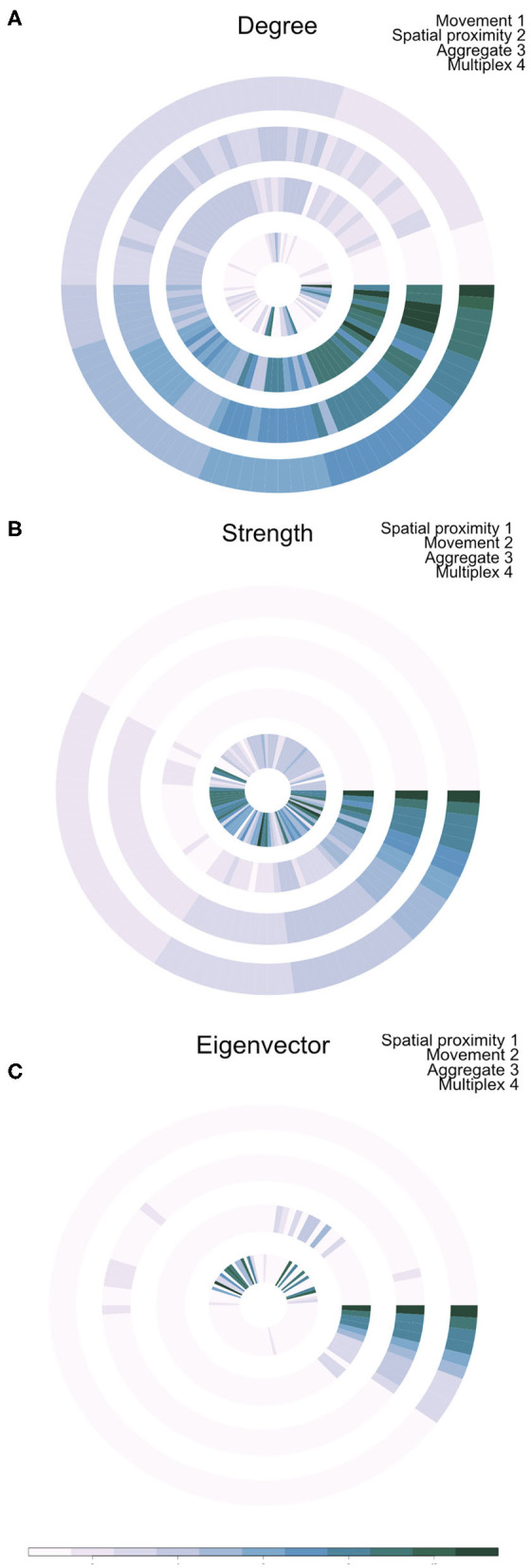
Annual visualization of node centrality metrics: **(A)** degree, **(B)** strength, and **(C)** Eigenvector centrality. Each node appears in the same position in each ring. Darker colors indicate nodes with higher centrality values. Each ring visualizes node centrality when measured in each layer separately (inner two rings), in the aggregate network (ring 3), and using the multiplex version of the metric (outer ring 4).

Outputs of this analysis were visualized as an annular plot in which a node appears in the same position in each ring of the plot (Figure 3). Figure 3A shows the annular visualization of node centrality for the subset of farms shown in Figure 2, with each segment representing a different node in the multiplex network and each ring representing the network layers. Across all three metrics shown here, it is clear that there is a variable correlation in centrality across the single-layer and multilayer networks (Spearman correlation coefficients range from −0.18 to 1.0, Figure 3). In particular, we see that farms with high strength or eigenvector centrality in the movement network are not necessarily the same farms that have high values of these measures in the spatial proximity network. For targeted disease control, the selection of key nodes based on a single layer could therefore be misleading.

Targeted disease control in livestock industries, especially as an outbreak response strategy, can also rely on defining control zones around infected premises, with strict control measures applied to farms within these zones. A related strategy, zonation, relies on defining regions of a country as disease-free for the purposes of international trade. An alternative approach to defining zonation and control zones called compartmentalization has also been proposed. A compartment is defined as a subpopulation of interlinked premises (such as a swine production system) with a common health status with respect to a specific disease, limited contact with premises outside the compartment, and for which surveillance, control, and biosecurity measures have been established for the purposes of trade (75). For a pathogen with multiple modes of transmission, it would be logical to define compartments based on connectivity in a multiplex network.

**Figure 4:**
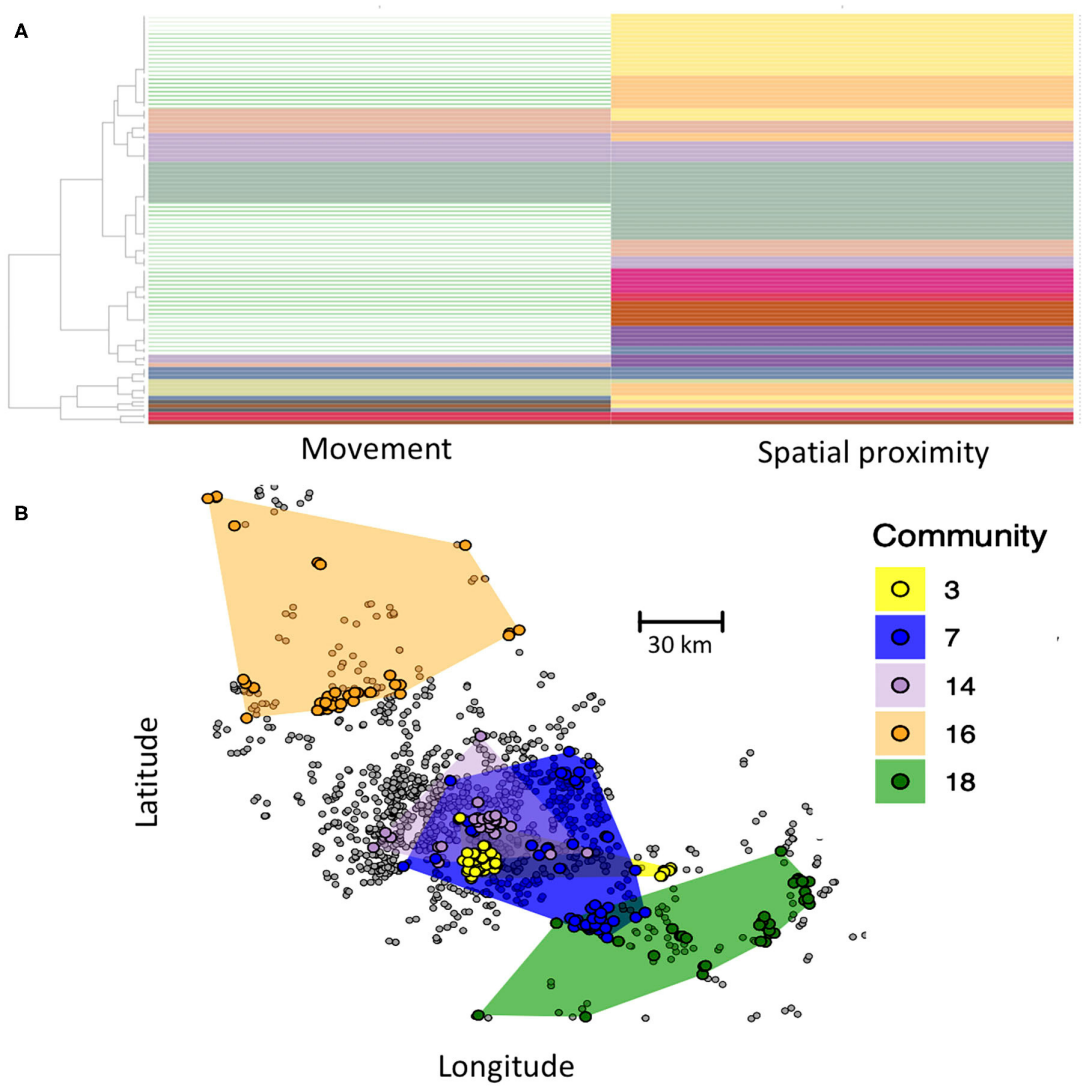
**(A)** Community structure of nodes based on Infomap community detection. Each row represents a farm, and each column represents its community assignment in the movement and spatial proximity networks, with some communities spanning both layers (same color in both columns). Rows with no color in the movement network are farms that only occurred in the spatial proximity network (no animal movements recorded) and thus did not have a community assignment in the movement layer. **(B)** Map of the five largest communities, with color of circles representing the community membership of farms. Spatial distribution of each community is shown by the colored polygons, which were determined by creating a minimum convex polygon around each community's farms. Farms in gray were not part of any of the largest five communities. Inter-layer edge weights were set to 0.01.

Here, we demonstrate the use of the Infomap multilayer community finding algorithm to define such compartments. Communities are thus defined as groups of farms that are in greater contact with one another than with farms outside of their communities. Critically, here contact between farms of the same community can either be through animal movement or spatial proximity. In the Infomap analysis, each node is assigned to a community in both the movement and spatial proximity layer, and some communities span both layers (Figure 4A). Our Infomap analysis identified numerous communities. If we map out the distribution of five largest communities in geographic space (Figure 4B), we see that each community generally includes several groups of farms that cluster tightly together in space, reflecting connectivity in the spatial proximity layer. However, different spatial clusters can occur within the same community if they are interlinked in the movement layer. This approach could thus be used to define groups of epidemiologically linked farms as compartments for pathogens with multi-modal transmission. From a disease control perspective, these compartments could be used to define high-risk (pathogen detected within the compartment) or low-risk farms (pathogen not yet detected in the compartment). Additional hypotheses could also be tested about transmission, such as the extent to which community membership influences pathogen diversity (i.e., do different communities have genetically distinct variants of a pathogen?).

## Important Considerations When Using Multilayer Networks

### Data Collection

Appropriate collection of network data is a fundamental challenge in the design of network studies, and it is important to ensure enough data is collected to provide a realistic insight into the study system (76–79). This problem is enhanced when using network modeling approaches in epidemiology, where missing edges can result in substantial underestimates of outbreak sizes (80). On the other hand, a similar but opposite problem might arise when the lack of a transmission-relevant contact data triggers the use of imprecise proxies such as shared contractors (i.e., two farms are considered connected when they use the same company for feed delivery, milk trucks, etc.): this could lead to overestimating the potential epidemic spread (13). For studies of infectious disease, it is typically necessary to construct networks over a time period relevant to the transmission of the infection of interest to increase the accuracy of network-based inference (81–83), which may provide time constraints on when data can be collected. Using multilayer network approaches can exacerbate this difficulty if it requires researchers to collect data on more different types of contact or interaction for multiplex networks, or potentially across more species or fomites for interconnected networks. Therefore, the feasibility of collecting sufficient data is an important consideration when weighing up whether to use multilayer approaches. Multilayer networks might be most naturally applied in wildlife epidemiology studies in which multiple different types of interactions between individuals are already recorded (84–86). In livestock context, while animal movements data have been regularly collected and analyzed in several countries in the past two decades (11, 57, 59, 87–89), challenges in adopting multilayer approach might arise for (i) countries where the collection of movement data and other industry-related information (e.g., farm location) is not mandatory, in particular developing countries (76), and (ii) including non-animal movements related potential infectious contacts, which data are often scarce and temporally limited (13, 90).

### Network Construction

There are also important considerations to be made when constructing multilayer networks for use in epidemiological studies. When layers consist of very different types of contacts or interactions involving distinct behaviors that are performed at different rates, it is possible that layers may differ drastically in their edge weights, and this can lead to problems with their analysis (16, 30). The same problem can also occur if sampling effort differs between layers. In multiplex networks with one layer that is much more well-connected than others, inferred transmission dynamics tend to be almost entirely controlled by the network structure of that single layer (91, 92). Therefore, in these contexts, it may be important to consider what added benefits using a multilayer approach can bring. If using a multilayer approach is still favored, then a variety of approaches are available to change the contribution of different layers (30) such as thresholding to produce unweighted or binary networks, or scaling/normalizing edge weights between layers (see our case study in this paper). In many situations, edge weights carry important information, especially when networks have a high density of connections, and incorporating edge weights can be important in network modeling of infection (93). As a result, any decision to threshold edge weights should be done with caution and be appropriate for the question being asked. A related consideration in multiplex networks is whether there is any redundancy between different layers (e.g., sets of intra-layer connections that are closely correlated with each other and represent the same set of ties). There are now a number of approaches available to calculate redundancy between layers in multiplex networks (94) that can provide valuable insights into the importance of taking a multilayer approach.

An important independent consideration in multiplex network studies is how interlayer edges should be weighted (16). While in interconnected networks, both intra- and interlayer network studies have natural weights, interlayer edge weights in multiplex networks are less intuitive as they typically connect the same actor to itself in different layers (whether this is an individual in a contact network or a farm in a livestock movement network). Epidemiological research offers an opportunity to provide interlayer edge weights with meaningful values in multiplex networks, especially when network modeling approaches are used. In these cases, interlayer edges can be used to represent the probability of being infected through one layer, causing an individual to be infectious in the second layer. For example, in a multiplex social network that included layers representing biting interactions (with knowledge that these could provide a transmission route) and close contact (as a proxy for aerosol transmission), interlayer edges could be weighted by the probability that an individual infected through being bitten by an infectious neighbor could subsequently transmit the infection through the contact network. A similar approach could be used for other multiplex networks (such as the between-farm networks in our case study). When these probabilities are unknown, then a sensitivity analysis on interlayer edge weights could be used to test the robustness of any conclusions drawn to these values, or alternatively, when empirical disease data is available, it might be possible to estimate these probabilities using an appropriately implemented network model.

### Points of Consideration

There remain some practical limitations in the analysis of multilayer networks (30). While methods for calculating descriptive metrics for multiplex networks have been widely developed and can be implemented using software packages in R (42) and Python (95), methods for the analysis of other multilayer networks (e.g., interconnected networks) are much less accessible. Therefore, when analyzing or modeling interconnected networks, it may be important to review the options available or feel confident in applying the calculations or algorithms required in the absence of ready-built functions. A similar consideration needs to be made when applying randomization-based analyses in multilayer networks. Especially for wildlife-based studies, comparison of multilayer networks to suitable permutations is likely to be important (30), and when conducting randomizations for multilayer networks it is important to consider both the research question being asked and any additional network features that arise as an outcome of the multilayer network structure of the data (17, 30).

## Opportunities for Future Use of Multilayer Networks in Veterinary Epidemiology

An exciting area for future application of multilayer networks in veterinary epidemiology includes the delineation of functional relationships between livestock systems for the implementation of subpopulation management strategies for World Organization for Animal Health (OIE)-listed diseases, as mentioned in the U.S. commercial swine industry example in a previous section. Establishing disease-free status throughout a country can be a difficult and timely undertaking. As such, the concepts of “zoning” and “compartmentalization” were developed by the OIE to recognize animals with different health statuses based on the geographical location (zoning) or based on management practices (compartmentalization) to facilitate the continuation of trade. A multilayer approach can be used to identify such subpopulations or estimate the risk of disease spread between subpopulations given different modes of transmission, which can guide the designation and maintenance of a subpopulation's disease-free status.

Moreover, multilayer networks can advance our knowledge of how temporal dynamics of network structure influence infectious disease spread. Contact networks of both wild and domestic animals are inherently dynamic (82, 96), and information contained in these contacts can change the rate of pathogen spread, as well as the efficacy of control strategies based on static networks. Many monolayer contact networks that incorporate temporality assume some level of aggregation of contacts over a period of time, such as calculating the mean or total edge weight for all edges over all time-steps, then use traditional techniques to characterize network properties (45). Others use traditional monolayer approaches to characterize network properties within each time-step then analyze the changes over the time-ordered layers (45). However, either approach results in loss of information and the ability to understand more complex interactions such as simultaneous interactions of multiple modes of contact and their evolution over time (97). It is prudent to note that the temporal analysis of an epidemiological process should consider the natural history of the pathogen under investigation in order to reflect the underlying epidemiological processes appropriately. Despite increases in research activity addressing the influence of temporality on complex systems, there is much to uncover regarding the impact of duration, concurrency, order of network properties, especially pertaining to disease transmission.

Multilayer network approaches are likely to be especially valuable at the (human-) livestock-wildlife interface (24), where identifying multi-host dynamics of pathogens is particularly important (98). Properly implemented multilayer network models will make it possible to better quantify the role of wildlife reservoirs of infection and estimate the rate of spillover from wildlife to livestock and vice versa. Taking these approaches may facilitate the identification of bottlenecks to transmission that can represent targets for management interventions or promote an understanding of the characteristic of individual animals or premises that play disproportionate roles in the spread or maintenance of infection in a community context. This extends naturally to encompass vector-borne transmission, especially for multi-host vector-borne diseases such as yellow fever, Lyme disease, and West Nile virus. A multilayer network approach could be used to unravel vital questions surrounding the management of such pathogens by incorporating factors that influence transmission, such as vector preference and host transmissibility.

## Conclusions

In this paper, we provide an overview of the early use multilayer networks in human and veterinary epidemiology. From the dynamics of coupled processes, such as information spread and disease transmission, to multi-host transmission, multilayer networks have been used to analyze a range of complex epidemiological systems that have been challenging to study in monolayer networks. Despite the caveats associated with their use, multilayer networks show promise in providing a powerful framework for furthering our understanding of the complex interactions that influence disease transmission dynamics in veterinary medicine.

## Author Contributions

AK, GR, MS, and KV contributed conception and design of the study and wrote sections of the manuscript. KV performed the analysis. All authors contributed to manuscript revision and approved the submitted version.

## Conflict of Interest

The authors declare that the research was conducted in the absence of any commercial or financial relationships that could be construed as a potential conflict of interest.
