# Supplementary material for: Multilayer and Multiplex Networks: An Introduction to Their Use in Veterinary Epidemiology
Source: Front Vet Sci. 2020 Sep 4;7:596. doi: 10.3389/fvets.2020.00596 (PMC7500177; doi:10.3389/fvets.2020.00596)
Supplement: Supplementary file 1 [file Data_Sheet_1.docx]

**Glossary**

***Aspect:*** characterization of a set of layers relating to the same feature or dimension (eg. time, space, infectious contact)

***Authorities/hubs***: nodes with highest (incoming/outgoing) centrality measure. Epidemiological context: high-risk nodes and super-spreaders.

***Betweenness centrality:*** the extent to which a fraction of the nodes in a network are important in connecting other nodes by their position on the shortest path in a network

***Centrality***: a measure of how central (i.e. important) a node is in the network

***Community***: a cluster of nodes more connections between them than with the rest of the network

***Compartmental models:*** a method used in mathematical modeling of infectious diseases in which the population is divided into discrete categories based on their infection status (i.e. susceptible-infectious-recovered, susceptible-latent-infectious-recovered, susceptible-infectious-susceptible)

***Connective redundancy:*** (1- neighborhood/total degree)

***Degree***: number of node’s connections. In case the network is directed we can define an in-degree, number of incoming connections, and out-degree, number of outgoing connections.

***Degree distribution***: distribution of the number of connections per node that is a helpful descriptor of network structure. Networks with heterogeneous (or heavy-tailed) degree distributions are susceptible to rapidly spreading epidemics.

***Eigenvector centrality:*** a measure of centrality that considers the importance of neighbor nodes

***Exclusive neighborhood:*** the number of nodes directly connected to a focal node only in that layer (or set of layers)

***Exclusive relevance:*** the percentage of neighbors present in a set of layers

***Infomap multilayer community finding:*** an algorithm for community-detection in multilayer networks

***Interlayer connections:*** connections between nodes in different layers of a multilayer network

***Intralayer connections:*** connections between nodes within the same layer of a multilayer network

***Modularity:*** a network property that measures the strength of a particular subdivision of a network. Often used alongside community detection algorithms to quantify the strength of the detected community structure.

***Multislice modularity maximization:*** an algorithm for community-detection in multilayer networks

***Network/graph***: a structure characterized by a set of objects in which some pairs of these are related. Epidemiological context: a population or a livestock system of interest.

***Node/vertex***: the fundamental unit of a network. Epidemiological context: individual animal host or group of hosts (farms, herds, sub-population, etc.).

***Link/edge***: a connection between two nodes. Epidemiological context: potential infectious contacts between nodes (face-to-face contacts between animals, animal movements between farms, etc.). Edges can be directed or undirected, weighted or unweighted.

***Giant strongly connected component (GSCC)***: a subset of the network in which any two nodes are reachable by following a directed path

***Giant weakly connected component (GWCC)***: a subset of the network in which all nodes can be reached when connections are bidirectional

***Interconnected/node-colored network***: multi-layer networks where different layers have different nodes. Epidemiological context: multi-species contact networks (cattle-sheep-pigs farms)

***Katz centrality:*** a centrality metric that accounts for the centrality of a node’s immediate neighbors

***Layer:*** a subsystem of a multilayer network that contains a set of nodes or edges characterised by a particular property (e.g. all male individuals or all aggressive interactions)

***Link density***: total number of contacts in the network (L) over the number of total possible contacts (N(N-1), where N is the number of nodes)

***Multidegree:*** a vector of the connectedness of a node in each layer of a multiplex network

***Multilayer network***: a collection of network layers connected by interlayer edges, which may contain multiple aspects

***Multiplex/edge-colored network***: multilayer where nodes in different layers are the same, while layers represent different types of connections. Epidemiological context: a different type of contact between animals (face-to-face and environmental contacts) or groups (animal movement and fomites contacts between farms); disease transmission and information dissemination networks between farms

***Multirelational network:*** a multiplex network in which each layer represents a different type of interaction

***Neighborhood:*** the nodes connected to a node in a layer or set of layers

***Network diameter***: the average shortest path in the network

***PageRank centrality:*** an indirect measure of centrality similar to eigenvector and Katz centrality that considers the number of connections that a node’s neighbors possess

***Relevance:*** the percentage of a node’s total number of neighbors present in a specified set of layers

***Shortest path***: the connection between two nodes through the lowest possible number of connections

***Spatial network***: a network where the presence of a link between two nodes is determined by the distance between those. Epidemiological context: wind-borne spread of highly contagious diseases (e.g. FMDv)

***Strength***: the sum of all node’s weighted connections in a weighted network. In case the network is also directed we can define an in-strength[out-strength], the sum of incoming[outgoing] connections

***Temporal network***: multiplex where different layers represent connections at different points in time

***Versatility***: a centrality measure computed in the context of a multilayer network, which takes into account the centrality of a node across all layers. It can be applied to multiple different centrality measures
